# Widespread pyocyanin over-production among isolates of a cystic fibrosis epidemic strain

**DOI:** 10.1186/1471-2180-7-45

**Published:** 2007-05-23

**Authors:** Joanne L Fothergill, Stavroula Panagea, Charles A Hart, Martin J Walshaw, Tyrone L Pitt, Craig Winstanley

**Affiliations:** 1 Division of Medical Microbiology and Genitourinary Medicine, University of Liverpool, Liverpool L69 3GA, UK; 2 Regional Adult Cystic Fibrosis Unit, Cardiothoracic Centre, Liverpool L14 3PE, UK; 3 Laboratory of HealthCare Associated Infection, Health Protection Agency, 61 Colindale Avenue, London, NW9 5HT, UK

## Abstract

**Background:**

Some isolates of the Liverpool cystic fibrosis epidemic strain of *Pseudomonas aeruginosa *exhibit an unusual virulence-related phenotype, characterized by over-production of quorum sensing-regulated exoproducts such as pyocyanin and LasA protease. Our aim was to determine the prevalence of this unusual phenotype amongst isolates of the epidemic strain, and to study other intraclonal phenotypic and genotypic variations.

**Results:**

The unusual phenotype was detected in at least one epidemic strain isolate from the majority of cystic fibrosis patients tested, and can be retained for up to seven years during chronic infection. Multiple sequential isolates of the epidemic strain taken from six patients over a period of up to nine years exhibited a wide range of phenotypes, including different antimicrobial susceptibilities. Our data suggest that each sputum sample contains a mixture of phenotypes and genotypes within the epidemic strain population, including within colony morphotypes. Many isolates exhibit premature (during early rather than late exponential growth) and over-production of pyocyanin, which has a number of toxic effects directly relevant to cystic fibrosis.

**Conclusion:**

The widespread occurrence of this unusual phenotype suggests that it may play an important role in the success of the epidemic strain.

## Background

With a carrier rate of 1 in 25 and an incidence of 1 in 2,500 live births, cystic fibrosis (CF) is the most common life-threatening, recessively inherited disease amongst Caucasians. During chronic pulmonary infection, CF patients suffer decline in lung function associated with periodic exacerbations triggered by bacterial products.

The most widespread CF pathogen is *Pseudomonas aeruginosa*, which causes infections that, once established, are impossible to eradicate.

The blue, redox-active phenazine pyocyanin is secreted by *P. aeruginosa *under the control of its cell-density-dependent quorum sensing (QS) system [1]. Pyocyanin and its precursor phenazine-1-carboxylic acid (PCA) have a number of toxic effects relevant to CF [2,3]. Pyocyanin and PCA inhibit the beating of human respiratory cilia in vitro [4,5], and alter expression of immunomodulatory proteins by human airway epithelial cells [6]. For example, increases in IL-8 and intercellular adhesion molecule-1 (ICAM-1) expression by oxidant-dependent mechanisms have been demonstrated in vitro and in vivo [6,7]. It has also been shown that pyocyanin production by *P. aeruginosa *induces neutrophil apoptosis and impairs neutrophil-mediated host defenses in vitro [8] and in vivo [9]. Furthermore, Kong *et al*. [10] have demonstrated that physiologically relevant concentrations of pyocyanin inactivate the airway vacuolar ATPase, resulting in reduced expression of CF transmembrane conductance regulator (CFTR) in cultured lung and nasal epithelial cells.

In 1996 Cheng *et al*. [11] used molecular typing to demonstrate the spread of a drug-resistant strain of *P. aeruginosa *[named the Liverpool Epidemic Strain (LES)] amongst patients in a children's CF centre in Liverpool, UK. Analysis of post-2000 patient samples identified the LES in 79% of 80 *P. aeruginosa*-colonised CF patients in the Liverpool adult CF centre, confirming the spread and longevity of LES infections [12]. Furthermore, a survey of 31 CF centres in England and Wales, involving the analysis of more than 1200 isolates of *P. aeruginosa*, identified the LES as the most common clone, being present in 48% of CF centres and accounting for 11% of the isolates [13].

In addition to its transmissibility, the LES appears to be more aggressive than other *P. aeruginosa *strains. It has been shown to replace previously established strains of *P. aeruginosa *(superinfection) [14], and it has infected both non-CF parents of a CF patient, causing significant morbidity [15]. In addition, there is evidence for greater morbidity amongst CF patients colonised with the LES compared to those carrying non-epidemic strains of *P. aeruginosa *[16].

Recently, we demonstrated that some LES isolates express an unusual phenotype characterized by the early and over-expression of QS-regulated virulence genes, including those encoding the secretion of pyocyanin, LasA and elastase [17]. The unusual phenotype was called hypervirulence because of increased virulence in a *Drosophila *infection model [17]. Here we refer to it as the over-production (of pyocyanin; OP) phenotype. We reported previously that this phenotype was detected in isolates from both non-CF parents and the CF patient of the unusual infection episode [15] but was lacking from an isolate (LES400) associated with a chronic infection [17].

In this paper, we determine the prevalence of LES isolates exhibiting the OP phenotype amongst adult CF patients in Liverpool, and report a wider analysis of LES isolates with respect to phenotypic and genotypic variations.

## Results

### Efficacy of a simple Luria broth test for pyocyanin production

**Figure 1:**
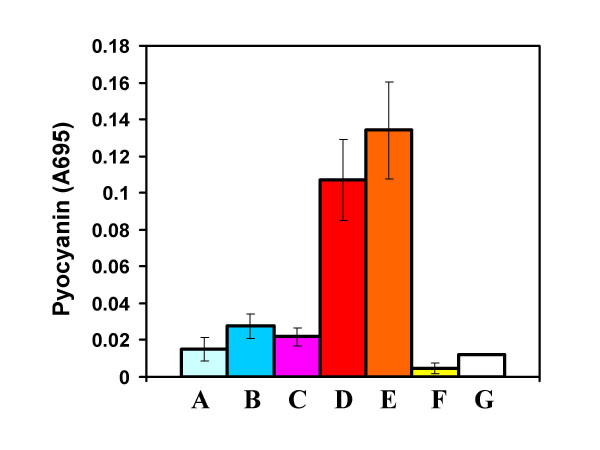
**Pyocyanin production in culture supernatants after overnight growth in Luria broth**. Mean absorbance values are shown for isolates of *P. aeruginosa *falling into the following categories: (a) water isolates (n = 10); (b) keratitis isolates (n = 10); (c) non-LES CF isolates (n = 29); (d) all LES isolates (n = 48); (e) LES isolates omitting known *lasR *mutants (n = 38); (f) LES known *lasR *mutants (n = 10); (g) strain PA01.

In order to test the efficacy of a simple screening procedure for pyocyanin production by strains of *P. aeruginosa*, the amount of pyocyanin in culture supernatants after overnight growth was quantified by measuring the A_695 _value for 10 environmental strains, 10 strains associated with bacterial keratitis, 29 non-LES CF and 48 LES isolates (Figure 1). Absorbance values for the supernatants of cultures of LES isolates were significantly higher than those for non-LES CF isolates (*p *= 0.004) or non-CF isolates (*p *= 0.015). When the known LES *lasR *mutants were removed from this analysis, the significance levels were increased further (*p *< 0.001 and *p *= 0.003 respectively).

### Prevalence of the OP phenotype

**Figure 2:**
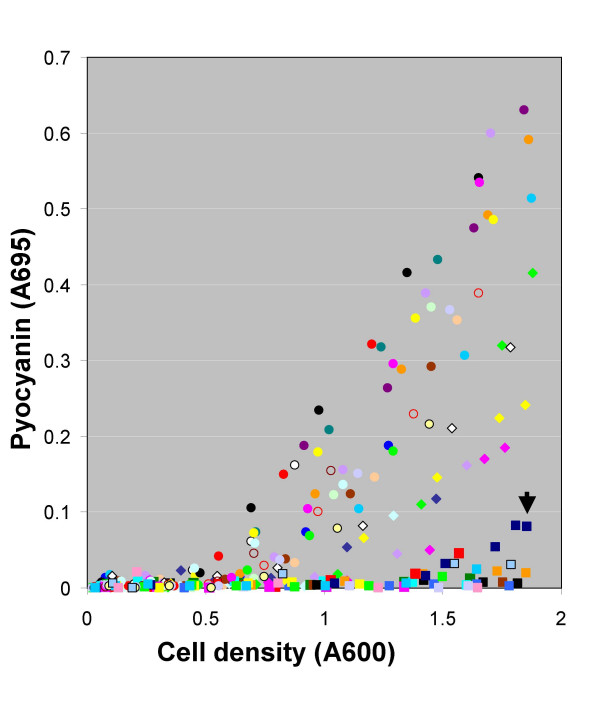
**Pyocyanin assays on LES isolates**. Production of pyocyanin in culture supernatants during growth of LES isolates in L-broth is shown for all the LES isolates tested in this study. Each combination of colour and shape plotted represents a different LES isolate. The only non-LES isolate included is strain PA01 (blue squares, indicated by the arrow). LES isolates could be sub-divided on the basis of pyocyanin assays into various groups: (i) OP (early- and over-production of pyocyanin) [circles]. (ii) Intermediate [diamonds]. (iii) Normal or negative [squares]

A total of 34 non-LES (each isolated from a different CF patient) and 55 LES isolates (isolated from 16 different CF patients and confirmed as LES by PFGE and/or PS21 PCR assay) of *P. aeruginosa *were screened for over-production of pigment by using the simple broth test. Overall, 51% of the LES isolates produced an increased blue/green coloration (above that of strain PA01) but 49% had normal or lower than normal pigment production. All of the non-LES isolates fell into the latter category. We selected all potential LES pyocyanin over-producers, and a selection of the other LES isolates, for further study using the full pyocyanin production assay. A wide range of pyocyanin production was observed (Figure 2). We grouped these activities as either (i) OP (pyocyanin A_695 _> 0.1 at cell density A_600 _= 1.2); (ii) Intermediate (pyocyanin A_695 _> 0.1 at cell density A_600 _= 1.7); (iii) Normal (pyocyanin detectable, similar to strain PA01) or Negative (no detectable pyocyanin activity). Using these criteria the LES isolates analysed could be separated according to pyocyanin production into 49% OP (n = 20), 17% Intermediate (n = 7), 34% Normal or Negative (n = 14). We did not sub-divide the latter two categories because of the difficulty in assigning those strains that did not reach equivalent cell densities to strain PA01. There was considerable variation in the amount of time taken for cultures of the different isolates to reach equal cell densities. Of the 20 CF patients from whom 1–6 isolates were subjected to study, 13 carried at least one OP isolate, and a further four carried at least one isolate of "intermediate" phenotype. For three CF patients, the isolates tested all had either a normal or a negative pyocyanin phenotype. We also screened six non-LES CF isolates using the full pyocyanin production assay. All fell into the categories Normal or Negative (data not shown).

### Variations in the OP phenotypes/genotypes amongst multiple isolates from individual patients

We identified six CF patients (patient A-F) from whom we had archived, multiple and sequential isolates of the LES (1995–2004). For each patient there was a minimum of four different LES isolates and three different sampling dates. There was considerable variation in the colony morphologies of the panel isolates. For each of these isolates both the pyocyanin activity and the LasA activity were assayed (Table 1). In addition, we determined the antimicrobial susceptibilities and, for the majority of isolates, assigned them to a LES pulsotype using PFGE (Table 1). There were widespread variations in both pulsotype and antimicrobial susceptibility data between isolates taken from the same patients.

We tested the panel of isolates for the common mutant phenotypes auxotrophy, lack of swimming motility and hypermutability. Six of the isolates (B55, B67, B41, 109, 49194, B48), from four different patients, exhibited the hypermutable phenotype. Only three isolates were unable to grow on glucose M9 media. These were all isolated from patient B (B46, 49194, B48) and two of the three were hypermutable isolates. All of the panel isolates were non-motile.

**Figure 3:**
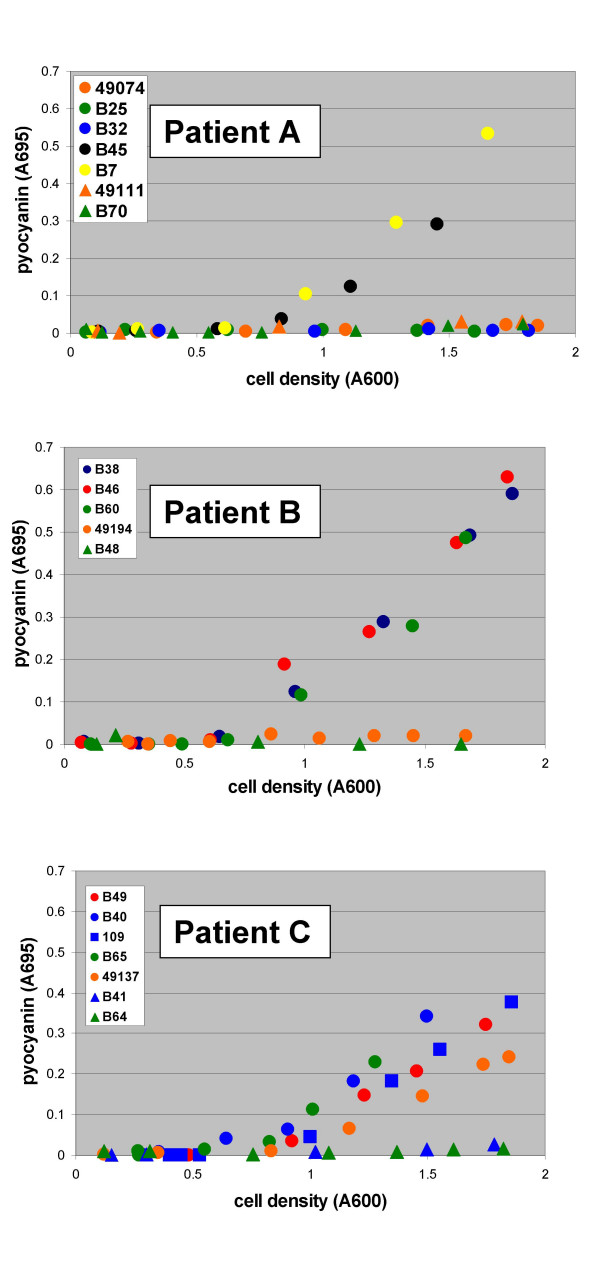
**Pyocyanin production by sequential isolates from CF patients A-C**. Production of pyocyanin in culture supernatants during growth of LES isolates in L-broth is shown for isolates from three CF patients (patient A-F).  Chronological order of isolation is indicated by colour from the earliest to the most recent in the following order : yellow (1995), red (1996), blue (1998), black (2000), green (2003) and orange (2004).  Different isolates from the same year are indicated by different shapes.

**Figure 4:**
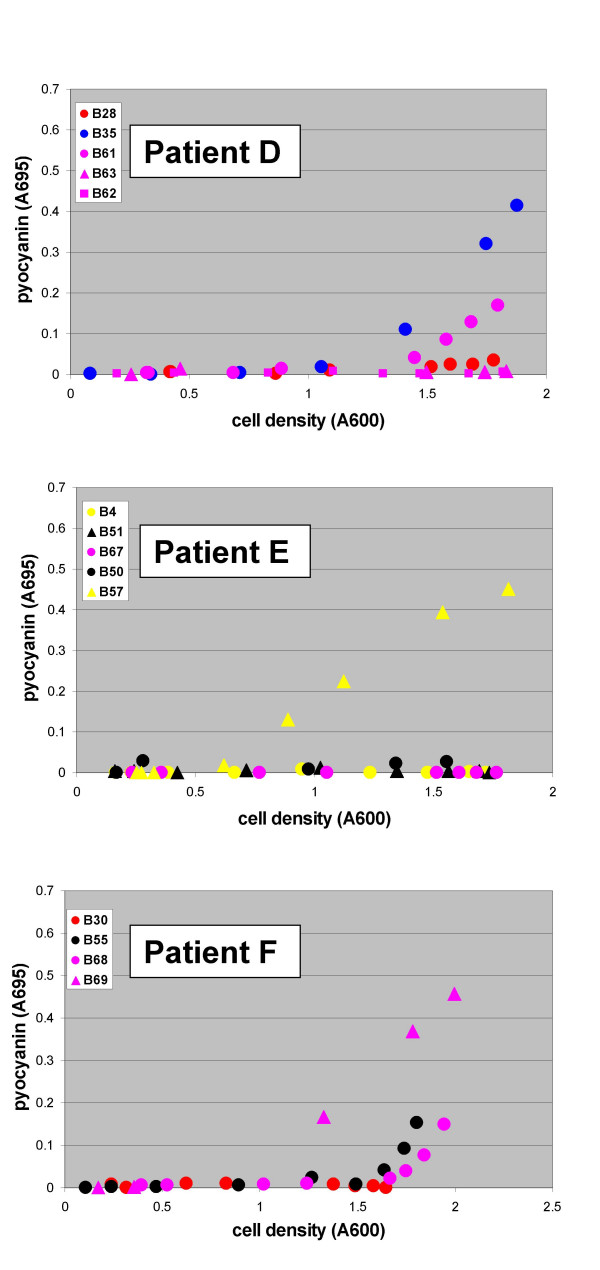
**Pyocyanin production by sequential isolates from CF patients D-F**. Production of pyocyanin in culture supernatants during growth of LES isolates in L-broth is shown for isolates from three CF patients (patient D-F).  Chronological order of isolation is indicated by colour from the earliest to the most recent in the following order : yellow (1995), red (1996), blue (1998), black (2000) and pink (2002).  Different isolates from the same year are indicated by different shapes.

Variations in pyocyanin production against cell density for LES isolates taken from patients A-F are shown in Figures 3 and 4. There was no general consensus in the results obtained. There was some evidence for temporal loss of the OP phenotype in patients A, B and E. The earliest isolate from patient A (from 1995) was of OP phenotype. However, the other isolate with the OP phenotype was isolated in 2000. Isolates from patient A included four identical non-OP *lasR *mutants, one of which was isolated in 1998. For patient B, early isolates were OP-positive whereas later ones were negative. For patient E, only one of two earliest isolates was OP-positive whereas all other isolates were OP-negative. For both patient B and patient E, two isolates from the same isolation time had different phenotypes with respect to pyocyanin production and LasA activity. This phenomenon could also be observed in patients C, D and F. However, isolates from both 1996 and 2003 from patient C had the OP phenotype. A similar longevity can be demonstrated with the data from patient B. In patient D, none of the isolates had a clear OP phenotype, whereas in patient F, the earliest isolate was non-OP whereas the most recent was OP-positive.

### Correlation between the OP phenotype and antimicrobial susceptibilities

Table 2 summarises the distribution of the OP-positive, OP-negative and other LES isolates according to resistance or susceptibility to five antimicrobial agents. According to the φ^2 ^test for statistical significance, in a comparison between isolates that were OP-positive and other isolates, there was a correlation between the presence of the OP phenotype and resistance to either ceftazidime (*p *= 0.006), aztreonam (*p *= 0.044) or meropenem (*p *= 0.037) (Table 2).

### *lasR *mutations are associated with loss of pyocyanin production

We analysed 17 of the pyocyanin-negative isolates for mutations in the *lasR *gene. Of these, *lasR *mutations were identified in 11 isolates (Table 3). As controls we also sequenced the *lasR *gene from five pyocyanin producers and confirmed that none were mutated (Table 3). Thus *lasR *mutations were significantly more frequent amongst pyocyanin-negative isolates (φ^2 ^test, *p *= 0.011). All of the *lasR *mutations that we have found in LES isolates to date are summarized in Table 3.

## Discussion

We have demonstrated that an unusual phenotype, characterised by early- and over-production of QS-regulated virulence factors such as the exoproducts pyocyanin and LasA protease, is widespread amongst CF isolates of the LES. In a simple broth test for pyocyanin production, whereby cultures were incubated overnight in 5 ml cultures, the levels of pyocyanin were lower than those measured during exponential growth in 50 ml cultures. It is likely that this was either due to some breakdown of pyocyanin or because of greater aeration during growth of the 50 ml cultures in 250 ml flasks. Hence, although the simple broth test is a useful screening tool, the full assay gives a better indication of actual levels of pyocyanin production.

A recent study sought to assess the genetic adaptation of *P. aeruginosa *strains during CF infections by whole genome analysis of sequential isolates [18]. The authors concluded that virulence factors required for initiation of acute infections are often selected against during chronic infection. Amongst those mutations leading to the loss of virulence-associated phenotypes, *lasR *mutations were the most common [18]. Indeed, the observation that *lasR *mutants are common amongst clinical and environmental isolates of *P. aeruginosa *has led to the suggestion that the QS system may have a negative impact on the organism's fitness [19]. If this is the case, then the unusual OP phenotype of the LES might be expected to amplify any negative impact. However, we found evidence that the OP phenotype can persist amongst isolates within a patient for periods in excess of five years (patients A, B and C) and up to seven years (patients B and C). To our knowledge there are no other reported instances of natural isolates of *P. aeruginosa *that exhibit the OP phenotype. Given that the OP phenotype places a considerable metabolic burden on the bacteria, the retention of such a high-maintenance phenotype within LES populations suggests that there may be some selective advantage. The fact that the OP phenotype is retained increases the possibility that it may play a role in the transmissibility of the strain. Interestingly, we also found that the same *lasR *mutant can persist within a patient for up to six years (patient A). We did not find complete correlation between pyocyanin over-production and high levels of LasA activity. This suggests that secondary mutations not affecting the whole QS regulon also lead to diverse phenotypes in LES populations.

CF isolates are often non-motile or auxotrophic. In one study, 33% of CF patients had a mean percentage auxotroph count of more than 50% total cfu/ml [20]. Oliver *et al*. [21] reported an overall prevalence for the hypermutable phenotype amongst CF isolates of *P. aeruginosa *as 20%. In our study, we found that LES isolates exhibiting the hypermutable phenotype comprised 18% of the isolates from the six CF patients. It has been suggested that the hypermutator phenotype plays an important role in the development of multiple-antimicrobial resistance in *P. aeruginosa *strains causing chronic lung infections [22]. However, we found little evidence that hypermutable isolates were less susceptible to antimicrobials than other non-hypermutable isolates from the same patient (Table 1).

Variations in colony morphology are also commonplace. Normal laboratory practice when dealing with sputum samples is to select a limited number of the most numerous colony morphotypes for storage, antimicrobial susceptibity profiling and occasionally strain typing. Morphology has been linked to increased virulence in the case of small colony variants (SCV) [23]. However, it has also been demonstrated that identical colony morphotypes sharing the same genotype can vary in activities such as antimicrobial susceptibility [24]. In our study, isolates obtained from an individual patient at the same sampling time differed in morphology, but there are a number of examples with sequential isolates where the same colony morphotype and genotype exhibited variations in antimicrobial susceptibity and/or QS-regulated activities. If pulsotype variation is discounted there are further examples suggesting that colony morphotype is an imperfect basis for the selection of isolate variants (Table 1). It has already been reported that the antibiotic susceptibilities of LES isolates vary widely [12,17].

The LES was first identified in a CF unit where ceftazidime monotherapy was being used as standard antipseudomonal treatment for respiratory exacerbations [11]. The authors suggested that the strategy of monotherapy had contributed to the success of the ceftazidime-resistant LES. Blázquez and colleagues [25] have not only suggested that ceftazidime concentrations thought previously to be lethal are not, but that ceftazidime treatment can increase the frequency at which *P. aeruginosa *mutates. Thus, it is entirely plausible that the strategy of ceftazidime therapy may have contributed to the emergence of the OP phenotype exhibited by the LES.

Although first identified because of the spread of a multiresistant clone in a CF unit [11], it is clear that isolates of the LES vary considerably in their antimicrobial susceptibility profiles. We found an interesting correlation between the OP phenotype and resistance to the β-lactams ceftazidime, aztreonam and meropenem. It has been demonstrated recently that pyocyanin can act as a signalling molecule in *P. aeruginosa *strains PA01 and PA14, upregulating in late exponential or stationary phase, a limited number of genes from the QS network, including the *mexGHI-opmD *genes involved in efflux, redox processes and iron acquisition [26]. We have reported previously that these genes are up-regulated in late exponential phase in the OP-positive isolate LES431 when compared to either an OP-negative LES isolate or strain PA01 [17]. Not only does this highlight another possible consequence of the early and over-production of pyocyanin in LES populations, but it suggests that pyocyanin itself may play a direct role in the observed link between antimicrobial susceptibilities and the OP phenotype.

Our data clearly support the notion that bacterial populations in the sputum samples of CF patients are mixed with respect to both genotype and phenotype, even when isolates represent the same clone. We saw many minor variations in PFGE banding patterns between isolates of the LES, even between isolates taken from the same sputum sample. The LES seems to have an unstable genotype [13,27], suggesting that individuals within LES populations undergo deletions, insertions and rearrangements. As well as these quite large genetic events, there are often point mutations leading to significant phenotypic changes. A study analysing greater numbers of isolates from each sputum sample may give a far better insight into the diversity within populations of *P. aeruginosa *in CF than the snapshot obtained when analysing relatively small numbers.

## Conclusion

Isolates of a CF epidemic strain of *P. aeruginosa *exhibit considerable phenotypic and genotypic diversity. The OP phenotype, characterized by de-regulation of the QS system leading to increased production of exoproducts including pyocyanin, is common amongst LES isolates and can persist within CF patients for several years. The presence and widespread nature of such an unusual phenotype amongst populations of a highly successful CF epidemic strain, suggests that the OP phenotype may play an important role in the success of the LES.

## Methods

### Bacterial strains

The bacterial strains used in this study were mainly isolates of *P. aeruginosa *from adults and children attending CF centres in Liverpool between 1995 and 2004. 34 non-LES isolates were obtained from 21 male and 13 female adult CF patients. The 55 LES isolates were obtained from 10 male and six female CF patients and included isolates from both adults and children over 14 years old. Of the LES isolates, 33 were from patients colonised with the LES for > 4 years whereas 22 were from patients colonised with the LES for < 2 years, including 15 from first-time LES-positive cultures. Similar data was not available for the non-LES isolates because strain-specific identification is carried out routinely only for the LES.

Ten *P. aeruginosa *strains associated with bacterial keratitis [28] and ten environmental water isolates [29] were used to assess levels of pyocyanin production by non-CF isolates. All strains were stored in glycerol-Luria broth at -80°C and cultured on Luria agar at 37°C. Strain PA01 [30] was used as a control.

### Phenotypic tests

Auxotrophy was tested by determining the ability to grow on glucose M9 minimal media. Swimming motility was assessed by inoculating bacteria on to the surface of 0.3% (w/v) agar plates containing 1% (w/v) tryptone and 0.5% (w/v) NaCl. The diameter of the swimming zone was measured after incubation at 37°C for 24 h. Each isolate was tested in triplicate. Hypermutability was assessed by determining spontaneous mutation rates on Luria agar containing rifampicin (300 μg/ml) following overnight growth in Luria broth as described previously [21]. Colony morphology was observed on MacConkey agar plates.

### Typing by pulsed-field gel electrophoresis (PFGE) and PS21 PCR assay

PFGE of *Spe*I- or *Xba*I-digested genomic DNA was carried out using 1% (w/v) agarose gels. Isolates with identical banding patterns were considered to be genetically indistinguishable and isolates with banding patterns differing by three or fewer bands to be clonal variants (pulsotypes) of the same strain. The PS21 PCR assay for identification of the LES has been described previously [31].

### Assays for QS-regulated phenotypes

Pyocyanin and LasA protease assays were carried out on culture supernatants during growth in Luria broth as described previously [17]. The amount of pyocyanin in culture supernatants was quantified by measuring the A_695 _value. For overnight cultures, it was important to shake the culture vigorously before obtaining the supernatant. This is to ensure that all of the pyocyanin present is in the oxidized form [32]. LasA protease activity was measured by determining the ability of *P. aeruginosa *culture supernatants to lyse boiled *Staphylococcus aureus *cells leading to a decrease in A_600_. Activity was expressed as the percentage of the initial (time zero) A_600 _value remaining after 60 min.

We used a simple broth test to identify strains with increased blue/green pigment production compared to strain PA01. Following overnight growth in universals containing 5 ml Luria broth, with shaking at 200 RPM, cultures were scored as either (i) greater pigment production than strain PA01 or (ii) similar or less pigment production than strain PA01. For validation of the test, pyocyanin production, as measured by A_695 _values in culture supernatants, was compared for various categories of *P. aeruginosa *isolates. Statistical significance was tested by using a one way ANOVA test (MINITAB 14.0, Minitab Inc.). Full pyocyanin production assays were carried out using 250 ml flasks containg 50 ml Luria broth.

### Identification of *lasR *mutations

PCR amplification was carried out as described previously [31]. Amplification of the *las *region was carried out using the oligonucleotide primer sets lasRF (5'- GTGCCGAATCCATATTTG-3')/lasRR (5'-CCTTCCCTATATATCTGC-3') and lasF1 (5'- GGGTATTCAGTTCGCATA -3')/lasR1 5'-CCCAAATTAACGGCCATA-3') to amplify overlapping regions which can be combined to cover the whole *lasR *gene. PCR amplicons were purified using S-400 microspin columns (Amersham-Pharmacia Biotech) and sequenced by Lark Technologies Inc. using the same oligonucleotide primers employed in the PCR amplification.

### Antimicrobial susceptibility tests

Antimicrobial susceptibility was assessed by disk diffusion tests according to BSAC guidelines [33] using disks (Oxoid) containing ceftazidime (30 μg), meropenem (10 μg), aztreonam (30 μg), tobramycin (10 μg) and ciprofloxacin (5 μg). Breakpoints for sensitive (S), intermediate (D) and resistant (R) organisms were according to BSAC guidelines.

## Authors' contributions

JLF and SP carried out most of the laboratory work leading to data acquisition. CAH, MJW and TLP participated in the design, supervision and coordination of the study, and in revision of the manuscript. CW participated in the design, supervision and coordination of the study, and drafted the manuscript. All authors read and approved the final manuscript.

## Figures and Tables

**Table 1:** Phenotypes and genotypes of sequential LES isolates from six CF patients

**Patient**	**Year**	**Isolate**	**PFGE**	**Pyocyanin (OP phenotype)**	**LasA{SD}**	***lasR *mut.**	**CAZ**	**MER**	**ATM**	**TOB**	**CIP**	**Colony morphology**
A	1995	B7	I-II	+	24.2 {4.9}	NO	R	R	R	D	D	SCV,NM,Ra,S
A	1998	B32	I-II	-	98.4 {7.2}	YES	S	R	S	D	S	SCV,NM,F,S
A	2000	B45	ID-II	-	67.3 {1.9}	nd	R	R	R	R	S	NM,F,S
A	2003	B25	I-II	-	98.6 {0.6}	YES	S	R	S	S	S	NM,Ra,S
A	2003	B70	IC-II	-	82.7 {5.1}	NO	R	R	R	S	S	M,Ra,S
A	2004	49111	ND	-	98.7 {1.6}	YES	S	R	S	S	S	NM,Ra,RW
A	2004	49074	ND	-	97.0 {1.5}	YES	R	R	R	S	S	NM,Ra,S
B	1996	B46	IH-IIE	+	81.6 {2.2}	NO	R	R	R	D	D	SCV,NM,F,S
B	1998	B38	I-II	+	84.8 {2.9}	NO	R	R	R	D	S	NM,Ra,S
B	2003	B60	IE-II	+	20.6 {1.9}	nd	R	R	R	D	S	NM,F,S
B	2003	B48	I-II	-	93.2 {0.9}	NO	S	R	R	S	S	M,Ra,S
B	2004	49194	ND	-	93.6 {0.9}	NO	S	S	S	S	R	NM,Ra,S
C	1996	B49	IC-II	+	43.3 {4.4}	nd	R	R	S	S	S	SCV,NM,F,S
C	1998	B40	I-II	+	20.3 {2.3}	nd	S	R	S	R	S	SCV,NM,F,S
C	1998	B41	I-II	-	98.6 {1.3}	YES	S	R	S	D	S	NM,Ra,S
C	1998	109	ND	+	82.7 {1.1}	nd	S	S	S	S	S	NM,F,S
C	2003	B64	I-II	-	95.2 {1.8}	NO	S	S	S	S	D	SCV,NM,F.S
C	2003	B65	I-II	+	8.61 {7.8}	nd	S	R	S	D	S	NM,Ra,S
C	2004	49137	ND	INT	63.4 {3.4}	nd	S	S	S	D	R	SCV,NM,F,S
D	1996	B28	IC-II	-	41.3 {5.5}	NO	S	R	S	S	S	SCV,NM,F,S
D	1998	B35	IC-II	INT	96.6 {1.2}	NO	S	S	S	D	S	SCV,NM,F,S
D	2002	B61	IC-II	NOR	3.33 {4.4}	nd	R	R	S	R	D	NM,Ra,S
D	2002	B62	I-IIG	-	95.3 {5.4}	YES	S	S	S	S	S	SCV,NM,F,S
D	2002	B63	I-II	-	98.5 {1.9}	YES	S	R	S	R	S	NM,Ra,S
E	1995	B4	IC-II	-	95.2 {2.0}	YES	S	S	S	R	S	NM,Ra,S
E	1995	B57	I-II	+	26.8 {3.2}	NO	R	R	R	S	D	NM,Ra,S
E	2000	B50	I-II	-	96.4 {3.6}	NO	R	R	R	S	S	M,Ra,S
E	2000	B51	I-II	-	98.4 {2.6}	YES	S	S	S	R	S	SCV,NM,Ra,S
E	2002	B67	ID-IIB	-	98.4 {2.9}	YES	R	R	R	S	D	NM,F,S
F	1996	B30	I-II	-	98.5 {1.0}	YES	S	S	R	R	S	SCV,NM,Ra,S
F	2000	B55	I-II	NOR	34.8 {4.4}	nd	S	S	S	S	S	M,Ra,S
F	2002	B68	IA-II	NOR	88.5 {4.4}	nd	S	S	S	S	S	NM,F,S
F	2002	B69	IB-IID	+	31.6 {5.6}	nd	R	R	R	R	S	NM,F,S

PFGE refers to pulsotypes obtained with *Spe*I and *Xba*I respectively; LasA activity indicates the percentage of the initial (time zero) A_600 _value remaining after 60 min; SD: standard deviation for four replicates; *lasR *mut. refers to whether the isolate is a confirmed *lasR *mutant or not by sequencing; ND: not determined; antimicrobial agent abbreviations are ceftazidime (CAZ), aztreonam (ATM), meropenem (MER), tobramycin (TOB) and ciprofloxacin (CIP); colony morphology was scored as mucoid (M), non-mucoid (NM), flat (F) raised (Ra), smooth (S), rough/wrinkled (RW), small colony variant (SCV). For OP phenotype: + indicates OP-positive; - indicates OP-negative; INT indicates intermediate; NOR indicates normal.

**Table 2:** Comparison between antimicrobial susceptibility and OP phenotype of LES isolates

Antimicrobial agent	% resistant LES isolates for different OP phenotypes		
	
	OP (n = 11)	Negative (n = 17)	N or I (n = 5)
CAZ R	73	24	20
			
ATM R	64	35	0
			
MER R	91	65	20
			
TOB R	27	24	20
TOB D	45	12	40
			
CIP R	0	6	20
CIP D	27	12	20

CAZ, ceftazidime; ATM, aztreonam; MER, meropenem; TOB, tobramycin; CIP, ciprofloxacin; R, resistant; D, intermediate antimicrobial susceptibility; N, normal; I, intermediate OP phenotype

**Table 3:** LES *lasR *mutants

LES isolate	*lasR *Mutation	Comments/Reference
LESB41	deletion of CCGGGCTGGCTACG leading to divergence from position R_66 _onward	isolated from patient C
LESB30	single point mutation leading to divergence from position S_33 _onward	isolated from patient F
LESB32	single nucleotide insertion/frame-shift mutation leading to divergence from position L_223 _onward	isolated from patient A
LESB25	same as LESB32	isolated from patient A
LES49111	same as LESB32	isolated from patient A
LES49074	same as LESB32	isolated from patient A
LESB63	same as LESB32	isolated from patient D
LESB62	single point mutation leading to single amino acid change H_224 _→ R	isolated from patient D
LESB4	same as LESB32	isolated from patient E
LESB51	same as LESB32	isolated from patient E
LESB67	deletion of AAGCGGAA leading to divergence from position A_134 _onward	isolated from patient E
LES49220	single point mutation leading to single amino acid change G_235 _→ D	CF isolate, isolated in 2004
LES400	repetition of GGTGCTC leading to divergence from G_123 _onward	[17]
LES430	deletion of GTGGATGCTC leading to divergence from position W_152 _onwards	[17]
LESB44	insertion of GAAG leading to divergence from position I_35 _onwards	[17]
